# Platinum Compound on Gold–Magnesia Hybrid Structure: A Theoretical Investigation on Adsorption, Hydrolysis, and Interaction with DNA Purine Bases

**DOI:** 10.3390/nano14242027

**Published:** 2024-12-17

**Authors:** Zhenjun Song, Mingyue Liu, Aiguo Zhong, Meiding Yang, Zhicai He, Wenmin Wang, Hongdao Li

**Affiliations:** 1 Engineering Research Center of Recycling & Comprehensive Utilization of Pharmaceutical and Chemical Waste of Zhejiang Province, School of Pharmaceutical and Chemical Engineering, Taizhou University, Taizhou 318000, China; liumingyue0820@126.com (M.L.); zhongaiguo@tzc.edu.cn (A.Z.); yangmd@tzc.edu.cn (M.Y.); hezhicai@tzc.edu.cn (Z.H.); 2 College of Chemistry and Materials, Taiyuan Normal University, Jinzhong 030619, China; wangwenmin0506@126.com; 3 Department of Chemistry and Chemical Engineering, Taiyuan Institute of Technology, Taiyuan 030008, China

**Keywords:** hydrolysis, guanine, gold–magnesia interface, platinum compound, density-functional theory

## Abstract

Cisplatin-based platinum compounds are important clinical chemotherapeutic agents that participate in most tumor chemotherapy regimens. Through density-functional theory calculations, the formation and stability of the inorganic oxide carrier, the mechanisms of the hydrolysis reaction of the activated platinum compound, and its binding mechanism with DNA bases can be studied. The higher the oxidation state of Pt (II to IV), the more electrons transfer from the magnesia–gold composite material to the platinum compound. After adsorption on the composite carrier, 5d←2p coordination bonds of Pt-N are strengthened. For flat and oblique adsorption modes of cisplatin, there is no significant difference in the density of states of the gold and magnesium oxide film, indicating the maintenance of the heterojunction structural framework. However, there are significant changes in the electronic states of cisplatin itself with different adsorption configurations. In the flat configuration, the band gap width of cisplatin is larger than that of the oblique configuration. The Cl-Pt bond range in the Pt(III) compound shows a clear charge reduction on the magnesia film, indicating the Cl-Pt bond is an active site with the potential for decomposition and hydrolysis. The substitution of chloride ions by water can lead to hydrolysis products, enhancing the polarization of the composite and showing strong charge separation. The hydrolysis of the free platinum compound is endothermic by 0.309 eV, exceeding the small activation energy barrier of 0.399 eV, indicating that hydrolysis of this platinum compound is easily achievable. ADME (absorption, distribution, metabolism, and excretion) prediction parameters indicate that hydrolysis products have good ESOL (Estimated SOLubility) solubility and high gastrointestinal absorption, consistent with Lipinski’s rule. During the coordination reaction process, there are significant changes in the distribution of frontier molecular orbitals, with the HOMO (highest occupied molecular orbital) of the initial state primarily located on the purine base, providing the possibility for electron transfer to the empty orbitals of the platinum compound in the LUMO (lowest unoccupied molecular orbital). The HOMO and HOMO-1 of the transition state and product are mainly distributed on the platinum compound, indicating clear electron transfer and orbital rearrangement. The activation energy barrier for the purine coordination reaction with the hydrolysis products is reduced to 0.61 eV, and the dipole moment gradually decreases to 6.77 Debye during the reaction, indicating a reduction in the system’s charge separation and polarization. This contribution is anticipated to provide a new theoretical clue for developing inorganic oxide carriers of platinum compounds.

## 1. Introduction

Oxide materials have been widely used in pollutant gas adsorption, pharmaceutical engineering, industrial catalysis, and energy conversion; the low-coordination sites and nanocrystalline structures, such as defects, doping, and hybrid structures, can have a significant influence on the properties and functions of oxides [1,2,3,4,5,6]. The charge transfer process on oxide surfaces often determines the redox and activation process of chemical bonds, as well as the generation process of free radicals of adsorbate molecules [7]. The construction of low-coordination sites of oxides can promote charge transfer [4,8,9] and generate irregular morphology such as defects, doping, heterostructures, and surface functional groups, which can enhance the chemical activity of inert oxide insulators (such as magnesium oxide). As a simple oxide with a rock salt structure, magnesium oxide is one of the most representative ionic crystals. MgO-based composites have attracted much attention due to their easy-to-obtain raw materials, stable mechanical properties, extraordinary electronic structure, and adsorption properties. Studies on the adsorption and heterogeneous catalysis of many oxide-supported metal systems have shown that the reactivity and selectivity are influenced by the electronic properties of the composite surface [10,11]. As a representative oxide insulator, MgO can be used to support transition metal nanoparticles and produce unique TM–Mg–O interface structures (TM refers to transition metals, especially gold and copper).

Compared with TiO_2_, MgO has a stronger adsorption binding interaction with Au_20_ clusters, which can lead to the formation of extended gold-plane clusters [12]. Gold ultrathin films formed by the epitaxial growth of gold on the surface of MgO films are key steps in the fabrication of top-down nanostructures with local plasmon resonance (LPR) [13]. Magnesium oxide–gold composites exhibit secondary electron emission (SEE) properties, which can be enhanced by doping elements in magnetron sputtering experiments [14]. The Au-MgO interface is considered to be a region of high catalytic activity for CO oxidation [15,16,17], and MgO can promote the formation of peroxide intermediate $\mathrm{CO}\cdot O_{2}$, where the peroxide exhibits a peroxide bond length of 1.45 Å and zero magnetic moment. Recent studies have shown that oxide morphology and structure can be regulated by the oxide nanoscale, such as thickness, substrate effect, or by doping transition metal with variable valence [18,19,20,21,22]. These structures are often regulated by increasing the basicity of the oxides, increasing the heat of adsorption, and activating Lewis acids through strong oxide–metal interactions (SOMI).

Magnesium oxide is a broad-band-gap oxide with generally limited chemical activity in the environment, and previous experimental and theoretical studies have confirmed that it requires more sophisticated and rational nanocrystallization to improve its adsorption reactivity [23]. The molecular hydrogen on the surface of pure magnesia is difficult to decompose, and it needs to absorb 1.84 eV of energy; specifically, the state of the homogeneous decomposition of hydrogen is endothermic by 3.06 eV. After the nanocrystallization of the magnesia catalyst, only 0.48 eV of energy is required for hydrogen decomposition, and only 0.79 eV of endothermic energy is required for homogeneous decomposition [7,24]. By constructing a Lewis acid–base pair on an oxide–metal composite, our group has recently obtained a thermodynamically favorable state of dihydrogen decomposition on oxide insulator film, exhibiting a very small activation energy barrier of 0.332 eV [25]. Pure perfect MgO has some ability to activate oxygen, but nanocrystalline MgO films can promote the formation of $O_{2}^{\cdot -}$ radicals, and surface distortions in the polarity of ultrathin oxides can stabilize free radicals [26,27].

Cheng et al. found that MgO film structures can also promote the adsorption and cleavage of hydrogen peroxide and organic peroxide (diethyl peroxide and acetone peroxide peroxyacetone) [28]. Diethyl peroxides and acetone peroxide exhibited positive dissociative adsorption energies on the surface of bulk magnesia, which were thermodynamically unfavorable, whereas, on the surface of MgO/Mo, they exhibited dissociative adsorption energies of −5.17 eV and −14.06 eV [28]. The interaction between water and oxide surface is a fundamental subject with important applications in the fields of photocatalysis, electrochemistry and sensors. Therefore, the catalytic reaction of water on the oxide surface attracts the attention of many scientists [29]. Xu et al. [30,31] proposed the strain-induced decomposition of water molecules on the surface of MgO. They studied the co-adsorption, activation, and decomposition of water molecules in the presence of molecular oxygen [27]. The thermodynamically favorable dissociation adsorption states (dissociative adsorption energy and activation energy (−1.231 eV, 0.733 eV) were obtained. Using cryogenic scanning tunneling microscopy (STM) experiments and density-functional theory calculations, Kim et al. revealed two water decomposition paths, which are electron excitation and vibration excitation on metal-supported magnesia films [32,33,34], and the corresponding calculated results show that the oxide–metal interface can be activated by ligand-field effect and interface defect.

The catalytic activation of common covalent bonds on oxide films has been widely studied, but the activation of coordination bonds of compounds on oxide films has been rarely investigated. Cisplatin-based platinum compounds are important clinical chemotherapeutic agents that participate in the majority of tumor chemotherapy regimens [35]. The antitumor properties of platinum-based drugs were discovered by chance by Rosenberg in his study of the mechanisms of how electric currents affect bacterial growth [36], and numerous subsequent studies have demonstrated that this drug can inhibit DNA replication and transcription and lead to cancer cell apoptosis [37,38,39]. Platinum-based anticancer drugs such as cisplatin have obvious toxic side effects, and after several rounds of cisplatin chemotherapy, magnesium levels in the blood of many patients are reduced to dangerous levels [40]. Low blood magnesium can cause a range of conditions, such as nausea, vomiting, anorexia, muscle weakness, seizures, and even coma. Platinum-based drugs are used in combination with magnesium oxide and are thought to reduce the neurotoxicity and nephrotoxicity of platinum-based drugs, as well as the prevalence of the hypomagnesemia caused by platinum-based drugs, with potential clinical value [41,42]. The combination of broad-band-gap oxides with noble metal additives of the IB subgroup element can increase the electron transition between the valence band and the conduction band [6], causing the O-2p electron state to move from the low energy level to the Fermi energy level, and enhancement of the absorption peak and electronic properties of oxides [43]. Pacchioni et al. [12,44] have studied the binding and nucleation mechanism of Au nanoclusters to MgO, and the differential charge density and electronic density of states indicate that gold nanoclusters can effectively bond to MgO. Sterrer et al. [45] predicted the binding mechanism of single-crystal MgO to two-dimensional gold nanostructures, with the existence on apparently polarized atoms at the interface between gold and magnesia, resulting in dipole-dipole interactions. Gold nanoparticles have good biocompatibility, non-toxicity, selective permeability and retention effect of tumor cells. Gold nanoparticles are widely used in photothermal and immunotherapy of cancer, and furthermore gold nanoparticles can be metabolized out of liver tissue [46,47].

In this contribution, the loading and activation effect of MgO ultrathin film on cisplatin, as well as the hydrolysis reaction and pharmaceutical reaction with DNA base, are investigated by density-functional theory calculations. The theoretical models and methodologies are illustrated in Section 2. The calculation results are discussed to reveal the formation and stability of gold–magnesia hybrid surface (Section 3.1), cisplatin adsorption behavior on Au (111) supported magnesia (Section 3.2), formation of Pt(III) compound on Au (111) supported magnesia (Section 3.3), cisplatin hydrolysis on Au (111) supported magnesia (Section 3.3), Pt(III) compound hydrolysis process (Section 3.5), interaction profile of Pt(III) compound with purine bases (Section 3.6) and influence of the temperature and the substituting group (Section 3.7). In the conclusion section, we summarized the key results of this contribution. As far as we know, the adsorption, activation, and hydrolysis behavior of platinum compound on usually inert insulating oxide (MgO) has never been reported before our group’s contribution. We anticipate that this contribution can provide theoretical clues and references for the experimental research and development of platinum drug carriers.

## 2. Methodologies and Models

The OptB88-vdW functional [48,49,50] is utilized to deal with the dispersion forces and exchange-correlation effect internally and between the magnesia–gold composite and adsorbates. The nonlocal functional conducts integral conversion from the double real space to reciprocal space to enhance the execution efficiency. Generalized gradient approximation (GGA), including nonlocal corrections used in this study, possesses more accurate superiority than standard local density approximation for computing complex interactions in the oxide-based composite. Exchange-correlation energy can be obtained by the following equation,

(1) $$E_{xc}=E_{xGGA}+E_{cLDA}+E_{cnl}$$

where *E_xGGA_*, *E_cLDA_*, and *E_cnl_* represent exchange energy and LDA/nonlinear correlation energy, respectively. The inner core electrons are treated by the PAW technique [51], which can reduce plenty of nuclear plane waves. An energy cut-off as large as 500 eV is set to ensure the accuracy of energetic computation. All the periodic density-functional theory calculations are carried out utilizing the Vienna Ab initio Simulation Package (VASP) [52,53].

The calculated lattice constant of magnesia (4.232 Å) agrees well with the experimental value (4.220 Å [54]). The lattice constant of gold metal adopts the experimental reported value of 4.078 Å [55]. Comparatively, the OptB88-vdW functional can produce more accurate structural and electronic properties than pure PBE functional, as shown in Tables S1 and S2. The optimized O-Mg distance and lattice constant are very close to the experimental values, while the pure PBE functional shows a larger deviation. The band gap at OptB88-vdW (5.118 eV) has an obvious advantage over that at pure PBE functional (4.506 eV). For the cisplatin compound, compared with the pure PBE functional, the computed bond and angle parameters at OptB88-vdW are closer to the parameters obtained with the hybrid functional.

A four-layer gold (111) substrate composed of (3 × 3) supercell is created along surface vectors [1 0 1] and [0 1 −1] with mesh lengths 17.303 Å, and then an ultrathin magnesia (111) film is deposited on the gold substrate. The large supercell surface is constructed to make a compromise between calculation time-demanding and the lateral interaction forces among adsorbed compounds. To match lattice vectors of gold (111) substrate, the supported magnesia is compressed by 3.63%. The influence of the thickness nanoscale of the gold substrate on the geometric structure (surface distortion) and d electrons of surface gold atoms are examined, as shown in Figure S1. The scenario for thicker two monolayer magnesia is examined (Figure S2), which will distort to form a much thicker magnesia film with substantially large surface rumpling (1.818 Å and 1.825 Å). The significant distortion of magnesia (111) agrees with the experimental metal-supported magnesia (111) morphology showing extended slight corrugation [56]. Thus, we prefer monolayer magnesia, which will bind more firmly to the substrate. The perfect ultrathin MgO is indeed challenging to synthesize in reality, and we adopt this ultrathin MgO to reflect a particular possibility for constructing a small amount of magnesia and a large amount of gold. According to previous experimental reports, a small amount of magnesia containing monolayer morphology could be obtained via real-time oxidizing submonolayer magnesium atoms on the metal substrate under ultralow oxygen pressure utilizing reactive molecular beam epitaxy as a function of the substrate temperature [56,57,58].

The bottom two layers of gold (111) substrate are fixed at their optimized bulk positions to maintain the properties of gold solids. The adsorbed cisplatin, water, magnesia film, and the top two layers of gold substrate are fully optimized with the residual force criterion 0.02 eV Å^−1^ satisfied. For K-point sampling of geometric optimizations, Γ centered Monkhorst-Pack grids (gamma only) are used, and denser grids (2 × 2 × 1) are applied to sample k points in the first Brillouin zone to integrate the static electronic properties. For energy minimization, the convergence criterion is 1.0 × 10^−5^ eV. The Gibbs free energy is defined as the following equation,

(2) $$G_{T}=H_{T}-TS=\Delta U\left(0\to T\right)+ZPE-TS$$

For the present study, we use the normal temperature of 298.15 K to compute the Gibbs free energy. The thermal correction $\Delta U\left(0\to T\right)$, zero-point energy (*ZPE*), and entropy (*S*) are obtained by analyzing frequency calculation results [59]. The negative interaction energy indicates an exothermic adsorption reaction. To separate periodically repeated slabs and refrain from image−image interaction, the imposed vacuum layer thickness is larger than 17 Å.

Potential energy diagram for hydrolysis reaction for platinum (III) compound on magnesia film and corresponding activation barrier are obtained utilizing the climbing image nudged elastic band (CI−NEB) technique implemented in the VTST program [60]. Bader charge analysis is carried out using the program developed by Henkelman’s group [60,61]. With delicate fast Fourier transform (FFT) grids, the charge population can accurately reproduce the core charge. The charge density gradient projection along path (*i*, *j*, *k*) can be calculated along the direction to neighboring points by the following equation,

(3) $$\nabla \rho \left(i,j,k\right)\cdot \hat{r}\left(di,dj,dk\right)=\frac{\Delta \rho }{\left|\Delta \hat{r}\right|}$$

where (*di*, *dj*, *dk*) is an integer vector standing for the step along the grid to the neighboring grid point [62]. The charge population analysis is generally qualitative, and we should pay attention to the charging transfer trends rather than the absolute charge values.

At the M06-2X/6-31G(d)/lanl2dz theoretical level of the Gaussian program [63], the transition state structure in potential energy diagrams for hydrolysis reaction and combination reaction between platinum compound and guanine base is located using the Berny algorithm. Under this algorithm, the following new estimate of the location of the stationary point is given by the equation

(4) $$x_{i}^{new}=\tilde{x_{i}}-\underset{j}{\sum }F_{ij}^{-1}\tilde{g_{j}}$$

where *F_ij_* is a second derivative matrix, and the derivative $\tilde{g_{j}}$ can be obtained from interpolation. Pharmacokinetics and drug-like properties can be predicted using the ADME (absorption, distribution, metabolism, and excretion) tool and density-functional calculation [64,65,66]. In this contribution, the solvent effect is not incorporated in the DFT methodology, and an explicit single solvent molecule (water) is added under different scenarios to investigate the hydrolysis reaction. The influence of the solvent effect on the geometric structure and charge population is shown in Figure S3, which indicates that the incorporation of the solvent effect can make the result differ to a very limited extent. The charge population trends, the coordination bond distances, and the direction for the dipole moment are essentially the same. The calculation results without solvent effect can also produce meaningful general trends. The human environment is a complex fluid containing inorganic salt, plasma, lymph, proteoglycan, lipids, and amino acids. The incorporation of the solvent effect could be artificial favoritism in the interaction force between water and platinum compound (or purine base). In contrast, the interaction forces between various inorganic ions (or organic materials such as plasma, lymph, proteoglycan, lipids, and amino acids) and platinum compounds (or purine bases) are asymmetrically neglected. The temperature effect for thermochemistry during coordination reaction is investigated employing the Shermo code [67]. Electronic and geometric structures for platinum compound adsorption and reaction on the gold–magnesia hybrid surface are further analyzed utilizing Visual Molecular Dynamics (VMD) [68], VASPKIT [59], Multiwfn program [69], and the VESTA program [70].

## 3. Results and Discussion

### 3.1. Formation and Stability of Gold–Magnesia Hybrid Surface

**Figure 1:**
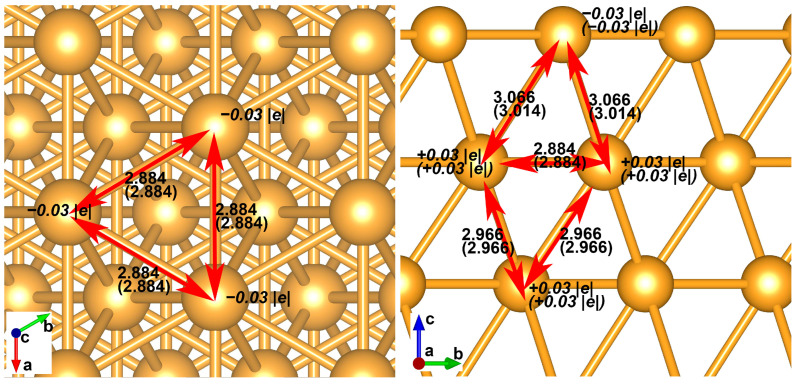
The structure and charge population of gold (111) film fully optimized with gamma point and (2 × 2 × 1) k-point meshing. The Au-Au distances and charges in parentheses correspond to the values obtained at denser (2 × 2 × 1) k-point meshing.

**Figure 2:**
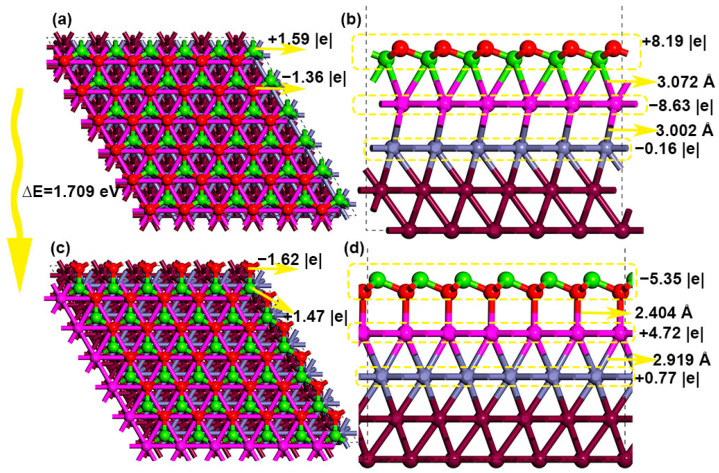
The structure and charge population of gold (111)-supported magnesia (111) film. (**a**,**b**) correspond to the stable equilibrium structure, while (**c**,**d**) correspond to the unstable structure with perpendicular gold-oxygen bonds. Green and red balls represent magnesium and oxygen atoms, and the pink, cyan, and brown balls represent top-layer, second-layer, and bottom-two-layer gold atoms.

Au(111) surface is optimized with gamma k-point meshing, and the results of charge population and bond distance agree very well with parameters obtained at denser (2 × 2 × 1) k-point meshing. The top atoms of Au(111) show negative charges (−0.03 |e|), while the gold atom of the secondary outer layer shows positive charges (+0.03 |e|). The surface gold atoms form gold triangles with an Au-Au distance of 2.884 Å, as shown in Figure 1. Surface gold forms an Au-Au bond (3.066 Å) with inner-layer gold atoms. The interlayer Au-Au bond distance between the second and third-layer gold atoms is shortened to 2.966 Å. The MgO-Au composite presents two distinct structural geometries when combined with magnesia (111) ultrathin film. First, the magnesium approaches the top gold atoms and forms the Mg-Au bond with a distance of 3.072 Å. The magnesia film is positively charged, with 8.19 |e| transferred to the gold slab. As shown in Figure 2, the surface magnesium carries a positive charge of 1.59 |e|, and the surface oxygen carries a negative charge of −1.36 |e|. The top-layer gold atoms grasp 8.63 |e|, and the second-layer gold atoms are only slightly charged (0.16 |e|). Second, the magnesia oxygen approaches the gold slab and forms an O-Au bond with a distance of 2.404 Å. In this case, the ultrathin MgO film is negatively charged to −5.35 |e|; the surface magnesium is 1.47 |e| charged, and the surface oxygen carries a negative charge of −1.62 |e|. Surface gold loses 4.72 electrons in total. In addition, the subsurface layer of gold loses 0.77 electrons. The interlayer Au-Au bond distance is obviously shortened to 2.919 Å. Due to the severe influence of charging and bonding of gold lattice, the second structural type with perpendicular O-Au bonding interaction is energetically unfavorable by 1.709 eV (Figure 2).

**Figure 3:**
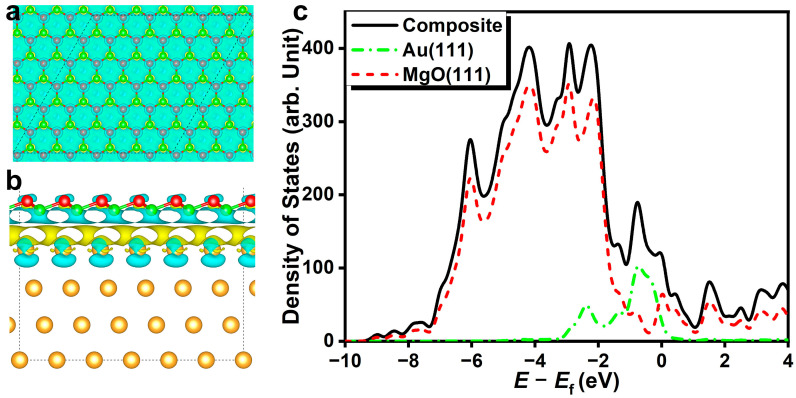
The differential charge density contour with charge density isosurface value of 0.001 e Bohr^−3^ (**a**,**b**) and localized density of states for gold (111)-supported magnesia (111) film (**c**). The yellow and cyan slices represent electron accumulation and electron depletion areas, respectively.

The differential charge density contour (Figure 3) indicates extensive charge transfer at the interface between the gold slab and supported magnesia films. Charge depletion occurs around interfacial magnesium atoms. The formation of a yellow strip at the interfacial area suggests sufficient charge accumulation, which facilitates the bonding interaction between magnesia (111) and gold slab. Charge depletion beneath top-layer gold atoms can be ascribed to the vast charge transfer to the interfacial regions. The inner-layer gold atoms do not exhibit a noticeable charge transfer effect, suggesting the interface structure formation does not damage the structural framework and bonding of gold bulk. Due to the steady ionic Mg-O bonds, for energy levels below −3.5 eV, most of the visible density of states can be assigned to magnesia (111). For energy levels between −3.5 eV to the Fermi level, gold and magnesia (111) exhibit overlapping densities of states, demonstrating the orbital hybridization of the interface structure. At the Fermi level, the magnesia film exhibits occupied electronic states, showing that the supported ultrathin (111) oxide film presents conductor properties, which differ significantly from the pristine insulating bulk oxide.

### 3.2. Cisplatin Adsorption on Au(111) Supported Magnesia

The flat adsorption state of cisplatin on Au(111) supported magnesia exhibits a slightly electron-accepting feature (−0.11 |e|, Table 1). The equilibrium structures for Pt(III) and Pt(IV) states show more negative total charges (−0.34 and −0.58 |e|). This result suggests that higher valence states of platinum lead to more electrons transferred from Au(111)-MgO composite to adsorbed platinum compound. The coordinated ammonia of free cisplatin carries a positive charge around +0.22 |e|. The flat adsorption configuration shows a more positive charging state +0.26 |e| of ammonia. Pt(III) state structure presents a positive charge (+0.25 |e|) for ammonia and a negative charge (−0.45 |e|) for the amidogen group. The higher valence state of platinum results in a more negative charging state of coordinated chloride ions, indicating higher ionicity of the adsorbed platinum compound. The valence state of platinum has limited influence on the charging state of Au(111) substrate but exerts an apparent effect over the charging state of magnesia (111) film. For the general trend, the high valence state of platinum brings about severe electron donation of magnesia film.

**Figure 4:**
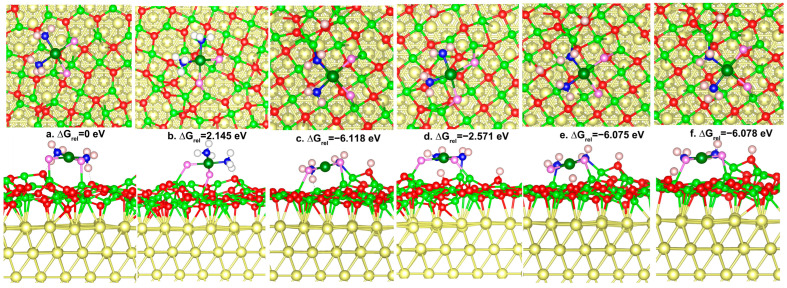
Adsorption and transformation of cisplatin compound on gold-supported 1 ML ultrathin magnesia (111). The Gibbs free energies are shown relative to the ground-state molecular adsorption state with flat adsorption configuration and Gibbs free energy of −380.373 eV. The white, blue, red, purple-, green-, cyan-, and yellow-colored balls stand for H, N, O, Cl, Mg, Pt, and Au, respectively.

The ground state for molecular adsorption exhibits flat adsorption configuration with Gibbs free energy −380.373 eV (Figure 4). Cisplatin combines with magnesia film through Cl-Mg bonds (distances 2.478 and 2.549 Å). The structural skeleton of cisplatin is maintained well. At the molecular adsorption state, the Pt-Cl bonds with distances 2.329 and 2.334 Å are longer than the isolated cisplatin compound (Table 2). However, the Pt-N bonds (2.061 and 2.066 Å) are shorter than the isolated cisplatin. The platinum ion forms a coordination bond with ammonia nitrogen, which results in the electron-donating effect from the 2p orbital of nitrogen to the unoccupied 5d orbital of platinum. The shortened Pt-N distance and the obviously decreased Bader charge of platinum ion indicate 5d←2p coordination bonds are strengthened when cisplatin is adsorbed. N-Pt-N and Cl-Pt-Cl angles are reduced to 94.7 and 92°, respectively, due to the adsorption behavior. The Gibbs free energy of the oblique adsorption configuration is 2.145 eV higher than the flat adsorption configuration. Cl-Mg distance is much longer (2.564 and 2.853 Å), indicating the interaction force between cisplatin and the composite is weakened. Due to weak interaction with the oxide film, the N-Pt-N and Cl-Pt-Cl angle (95 and 93°) is larger than the flat adsorption configuration. The oblique adsorption configuration grasps 0.097 electrons from ultrathin oxide film with ammonia positively charged (+0.25, +0.26 |e|), platinum positively charged (+0.47 |e|), and chloride negatively charged (−0.53, −0.56 |e|). The oblique adsorption configuration (Figure 4b) can be considered to be the transitional structure from free cisplatin to flat adsorption configuration. Due to the misalignment of the positive and negative charge centers, the cisplatin presents molecular polarity. When free cisplatin approaches the oxide film, the chloride with strong polarity should first interact with the oxide surface, bringing about the oblique adsorption configuration. Then, ammonia forms hydrogen bonds with surface oxygen, producing a flat adsorption structure.

**Figure 5:**
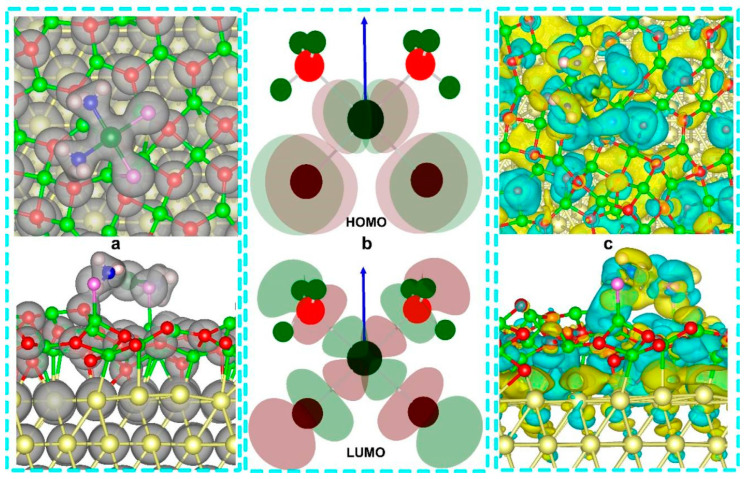
Charge density contours with isosurface value 0.05 e Bohr^−3^ (**a**), highest occupied molecular orbital and lowest unoccupied molecular orbital of cisplatin molecule (**b**), and differential charge density contours (**c**) with isosurface value 0.001 e Bohr^−3^. For differential charge density, the isosurfaces colored in turquoise and dark yellow represent charge accumulation and depletion, respectively.

**Figure 6:**
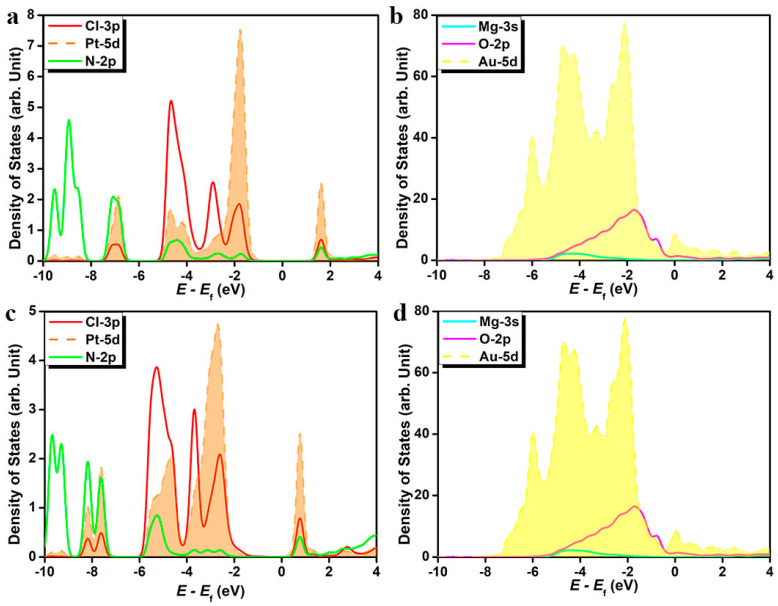
Localized density of states of platinum central ion, coordinated ammonia nitrogen, chloride ions, magnesium, oxygen, and gold slab for flat adsorption configuration (**a**,**b**) and oblique adsorption configuration (**c**,**d**).

Due to the maintenance of coordinate-covalent bonding inner cisplatin, continuous charge density contours distribute around the area between ammonia nitrogen and platinum and around the area between chloride and platinum (Figure 5). Charge density contour does not appear between surface oxygen and magnesium, suggesting the maintenance of ionicity of the O-Mg bond. A consecutive charge density contour appears between ammonia nitrogen and the neighboring surface oxygen. The HOMO contour mainly distributes in platinum and coordinated chloride ions. The participating atomic orbitals are the d orbital of platinum and the p orbital of chloride, and the HOMO produced by the linear combination of Pt-d and Cl-p is a π bonding orbital with symmetry consistency. The frontier orbital HOMO does not distribute in coordinated ammonia, indicating the firmness of the N-Pt dative bond, and the ammonia appears hardly able to serve as leaving group. LUMO contour spreads over cisplatin, and the p orbital of chloride and the d orbital of platinum form an antibonding σ orbital. Meanwhile, the N-p orbital and Pt-d orbital combine head to head to create another part of the molecular antibonding σ orbital. The differential charge density indicates widespread charge transfer between the adsorbate cisplatin and the hybrid composite surface. For flat and oblique adsorption, the densities of states of gold and magnesium oxide films do not differ significantly between the two cisplatin adsorption modes, indicating retention of the geometrical framework of heterostructure (Figure 6). However, the electronic states of cisplatin changed obviously when the adsorption structure differed. Under the flat configuration, the band gap width from the top of the valence band to the bottom of the conduction band of cisplatin is larger than that of the oblique configuration. The oblique configuration shows more density-of-state peaks of Cl-3p, indicating that chloride is bonded to multiple surface magnesium.

### 3.3. Formation of Pt(III) Compound on Au(111) Supported Magnesia

**Figure 7:**
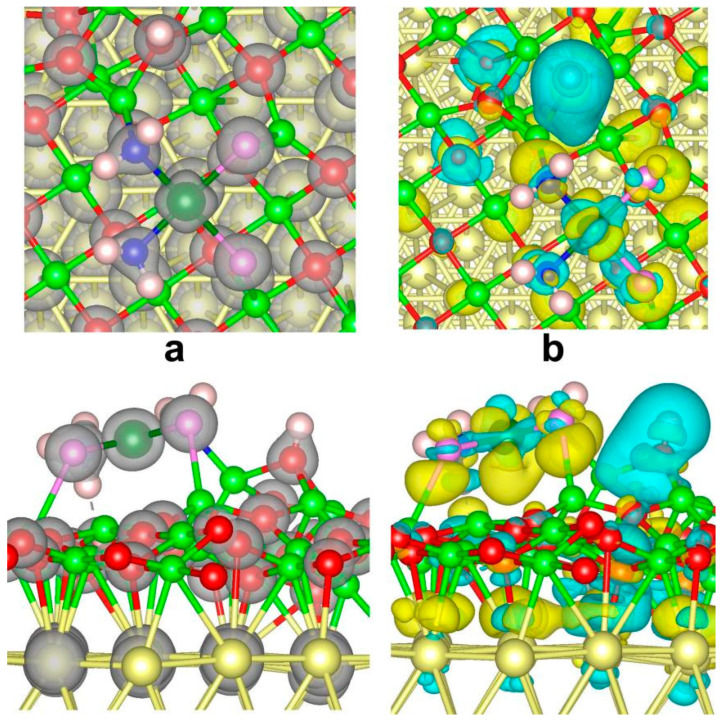
Charge density contours with isosurface value 0.15 e Bohr^−3^ (**a**) and differential charge density contours with isosurface value 0.003 e Bohr^−3^ (**b**) for Pt(III) compound adsorption on gold-supported magnesia (111) film. The isosurfaces colored in dark yellow and turquoise represent charge accumulation and charge depletion, respectively.

The oxide film can abstract one hydrogen from the coordinated ammonia, and the generated amidogen group binds more strongly with the central platinum ion with N-Pt distances of 2.073 and 2.055 Å (Table 2). The N-Mg bond of amidogen shows a distance of 2.127 Å. Meanwhile, the chloride ion binds with surface magnesium with distances of 2.492 and 2.658 Å. The dissociated hydrogen binds with surface oxygen at a distance of 0.973 Å. The continuous charge density contour between nitrogen and central platinum is maintained. From the differential charge density contour (Figure 7, defined as $\;\rho =\rho \left(\mathrm{tot}\right)-\rho \left(\mathrm{Au}\right)-\rho \left(\mathrm{MgO}\right)-\rho \left(\mathrm{cisplatin}\right)$), the surface magnesium loses electrons and the coordinative chloride abstracts electrons. The charge accumulation with yellow color and charge depletion with cyan color occur on chloride and below surface magnesium, respectively. Surface hydrogen tends to present charge depletion. The intermediate region between coordinative NH_2_ and surface magnesium shows a significant yellow color, indicating charge accumulation. The hydrogen of coordinative ammonia generates charge reduction, while the charge accumulation occurs on surface oxygen beneath the ammonia hydrogen, indicating considerable hydrogen-bonding interaction. Noticeable charge depletion occurs within the range of the Cl-Pt bond, suggesting the potential decomposition and hydrolysis of this Cl-Pt active site.

### 3.4. Cisplatin Hydrolysis on Au(111) Supported Magnesia

**Figure 8:**
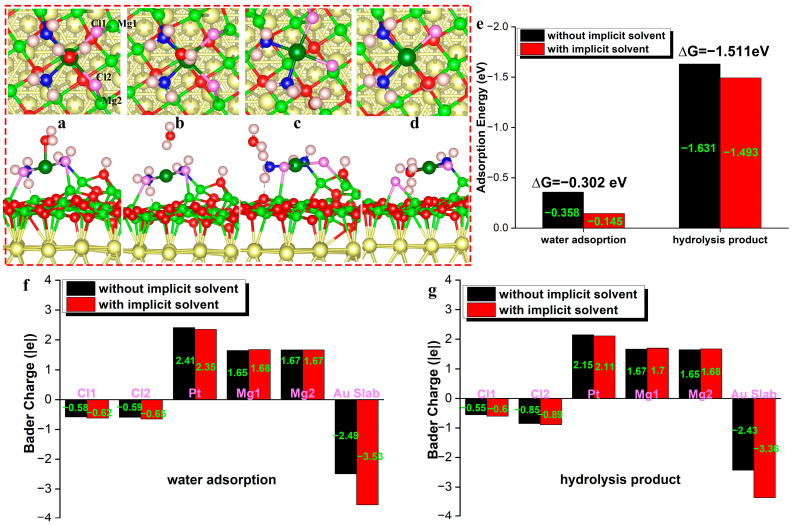
The hydrolysis of adsorbed cisplatin occurs gradually by water substitution. The assumed structure for water adsorption with oxygen linked with platinum (**a**), the optimized structure for water adsorption with hydrogen linked with platinum (**b**), water adsorption on ammonia (**c**), the water substitution structure (**d**), the water adsorption energy (**e**) and Bader charge for structural sites of water adsorption on platinum (**f**) and hydrolysis product (**g**).

After the reduction of the central valence state of cisplatin, the Pt(III) compound can stably adhere to the gold-supported MgO films. The water molecule is located directly above the platinum central atom (Figure 8a), and the adsorption energy is calculated to be −0.323 eV. The adsorption energy is calculated by the formula

(5) ΔGads=Gtot−GPtIII−MgOAu−GH2O

where *G*(tot) is the total Gibbs free energy of the system and GPtIII−MgOAu is the Gibbs free energy of Pt(III) compound adsorbed on gold-supported magnesia film.

The adsorption structure is also considered for the direct adsorption of platinum by water oxygen. This structure with an oxygen atom linked to platinum is unstable and transforms into a more stable structure with a hydrogen atom connected to platinum. The calculation demonstrates that the coordination number of platinum compound adsorbed on the magnesia surface is hard to change, presenting a tetra-coordinated structure where additional water molecules are challenging to coordinate in an augmented manner. When a hydrogen atom is attached to platinum (Figure 8b), the adsorption configuration exhibits an H-Pt distance of 2.531 Å. Each Cl-Pt bond is only extended by about 0.003 A, indicating that the water molecule interacts weakly with the platinum central atom. Water molecules can also come in close proximity to liganded ammonia molecules to form hydrogen-bonding interactions (Figure 8c). The water molecule close to the ammonia molecule presents an adsorption energy $\Delta G_{ads}\;$ of −1.322 eV, exhibiting a H_water_-N hydrogen-bonding interaction (1.879 Å). The ammonia molecule has a hydrogen-bonding interaction with the surface oxygen (1.735 Å), longer than the initial hydrogen-bonding bond length. The bonding of the ammonia molecule ligand with the central atom Pt is strengthened with reduced N-Pt distance. Adjusting the Pt coordination structure and forming the hydrogen-bonding interaction results in a significant energy decrease of the 8c structure.

The ammonia hydrogen with hydrogen bonds presents a positive charge of 0.49 |e|, while the charge of ammonia hydrogen without hydrogen bonds becomes significantly smaller (0.39 |e|). This charge transfer is offset by the electron-stripping effect of chlorine so that water molecules close to ammonia are generally neutrally charged (−0.002 |e|). Due to the coordination between N and the central platinum atom, ammonia has a positive charge of +0.27 |e|. The chloride ion close to the water hydrogen has a negative charge of −0.57 |e|, while the chloride ion far away from the hydrogen is more ionic and exhibits a higher charge (−0.61 |e|).

The hydrolysis product of the Pt(III) compound is formed when the water molecule replaces the chloride ion (Figure 8d). The oxygen atom coordinates with Pt to form a Pt-O bond of 2.046 Å. The coordinating water molecule is simultaneously bonded to the surface oxygen, with Os-H and Ow-H distances of 1.047 and 1.508 Å. The Cl-Pt bond distance is 2.334 Å, shorter than that before the substitution reaction (2.375 Å), whereas the coordinating chloride ion shows much weaker bonds with surface magnesium (Cl-Mg is lengthened from 2.56 Å to 3.129 Å). Platinum of the hydrolysis product exhibits a positive charge of 0.50 |e|, which is larger than the Pt of the hydrolyzed initial structure formed by the water molecule approaching the coordinated ammonia molecule (0.46 |e|). The charge value of the MgO film increases from 2.52 |e| to 2.89 |e| in the initial structure, and the number of electrons stored in the Au substrate rises from 2.2 to 2.43, indicating that the MgO-Au composite has enhanced polarization after hydrolysis and shows a strong charge separation. After hydrolysis, the whole water molecules change from negatively charged to positively charged (0.10 |e|), while ammonia presents a reduced positive charge of 0.25 |e|. Compared to the initial structure, the dissociated chloride ion presents a significantly enhanced negative charge of −0.85 |e|, which is related to the improved ionicity of the chloride ion.

The implicit solvent is imposed for the configurations for water adsorption on platinum to examine the influence of the implicit solvent effect. The Bader charges of surface magnesium, the coordinated chlorine, and the central platinum do not show apparent changes after the implementation of the implicit solvent model (Figure 8f,g) at both the water adsorption state and coordination hydrolysis state, indicating the influence of the implicit solvent model is quite limited for the charge transfer. As for the relative energy, the implicit solvent model also introduces substantially minor differences (Figure 8e). The reaction energy from the adsorption structure (ΔG for adsorption: −0.302 eV) to the hydrolysis structure is calculated to be −1.631 eV (ΔG=−1.511 eV) and −1.493 eV, respectively, demonstrating that the reaction thermodynamics without implicit solvent or with implicit solvent should be similar and show the same tendency. The calculation results imply that the interaction effect caused by the implicit solvent model should be much weaker than the chemical bonds between the surface and the reactants in the particular theoretical model.

### 3.5. Pt(III) Compound Hydrolysis Process

**Figure 9:**
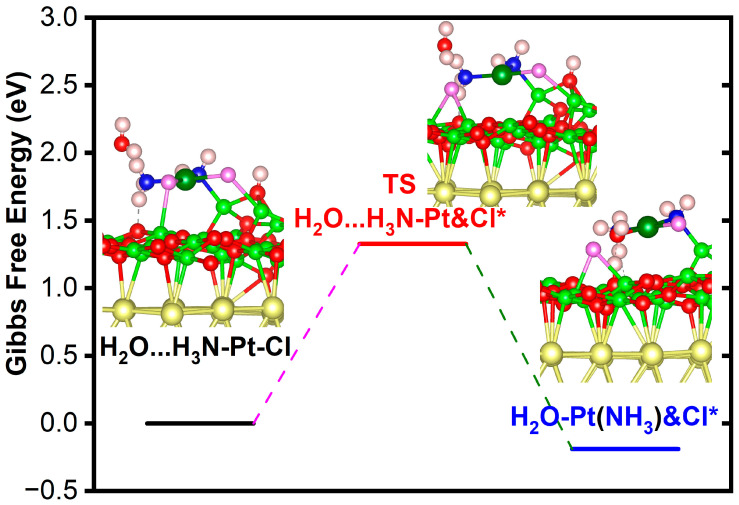
Potential energy diagram for hydrolysis reaction for Pt(III) compound on magnesia film. Structural models show relaxed structures for water adsorption on ammonia ligand (initial state), transition state, and water substitution product. The star * represents adsorption site.

The Pt(III) compound in the doublet state is −1.709 eV lower in Gibbs free energy than in the quartet state. Mulliken charge analysis shows that the Pt central atom of the free doublet state molecule is charged by 0.39 |e|. In contrast, the coordinated ammonia molecule and amino group are charged by 0.23 and −0.15 |e|, respectively. Compared with the adsorbed state, the charge of the amino group is significantly reduced. The reaction route and transition state of the Pt(III) compound during the surface hydrolysis process are shown in Figure 9. This is a chemical reaction process with a slight decrease in Gibbs free energy (−0.189 eV), requiring a Gibbs free energy barrier of 1.328 eV to pass through. To accurately consider the hydrolysis reaction characteristics of the Pt (III) compound, we verified the hydrolysis reaction between ground-state free Pt (III) compound and water molecules through quantum mechanical calculations at the M06-2X theoretical level. The free Pt(III) compound forms hydrogen bonds between ammonia hydrogen and water oxygen, forming $H_{2}\mathrm{NH}\cdots {\mathrm{OH}}_{2}$. The length of H_am_-O_w_ is 1.847 Å. The HOMO-LUMO energy gap of the reaction system is 7.739 eV, rendering it difficult for electrons to transition to high-energy orbital. The HOMO-LUMO energy gap of the transition state is decreased by 0.969 eV. In the transition state, the water molecule bonds with the Pt central atom, with a bond length of 2.059 Å. The coordination of water molecules in the transition state structure does not affect the coordination number of Pt (it is still a tetracoordinate structure), and the substituted chloride ion is no longer coordinated with platinum (another chloride is still coordinated with platinum).

**Figure 10:**
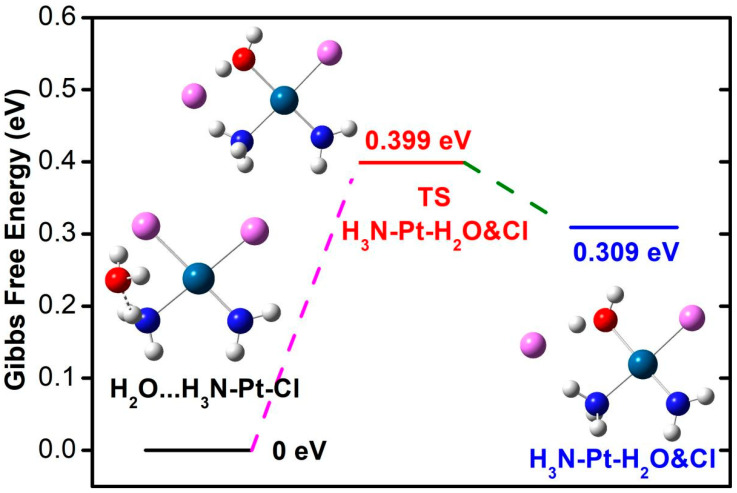
Potential energy diagram for the hydrolysis reaction in the first step for a free Pt(III) compound. Structural models show relaxed structures for water adsorption on ammonia ligands (initial state), transition state (TS), and water-substituted product.

**Figure 11:**
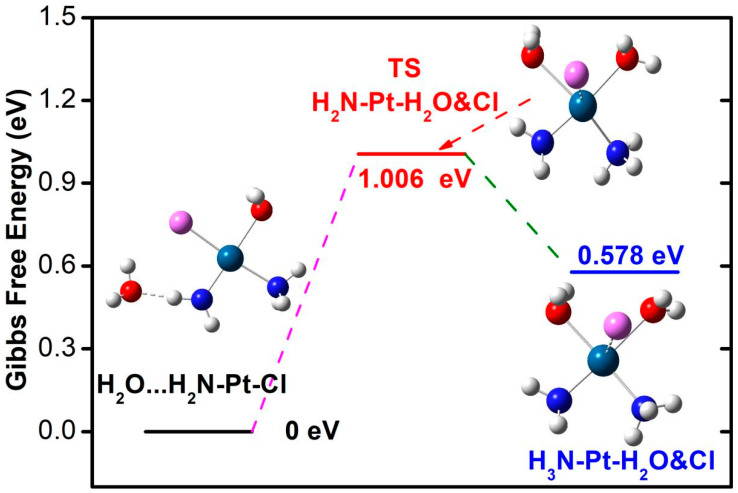
Potential energy diagram for second step hydrolysis reaction for free Pt(III) compound. Structural models show relaxed structures for water adsorption on ammonia ligand (initial state), transition state (TS), and water-substituted product.

The hydrolysis reaction product is conformationally closer to the transition state, and the structure undergoes further adjustment, with the shortening of the coordination bond distance of the amino and ammonia with platinum. The coordination bond between the water/chloride and platinum is lengthened compared to the transition state. The Gibbs free energy change image of the hydrolysis reaction is shown in Figure 10, with a reaction heat uptake of 0.309 eV and a transition state energy barrier of 0.399 eV to be crossed, indicating that the hydrolysis of platinum (III) compound is easy to achieve. Calculation of the secondary hydrolysis of the Pt(III) compound suggests that the reaction is less kinetically viable than the primary hydrolysis (Figure 11). The secondary hydrolysis reaction is energetically endothermic by 0.578 eV and presents an activation energy barrier of 1.005 eV in the scheme of Gibbs free energy. The computed energetics are consistent with previous experimental and theoretical results, indicating that the mono-aquated form is more favorable than the fully hydrated form. In addition, the ADME parameters, pharmacokinetics, and drug-like properties of the energetically feasible hydrolysis product are preliminarily predicted using the swissADME tool [64]. ESOL (Estimated SOLubility) is predicted to be 75.4 mg/mL, classified as very soluble. Pharmacokinetics shows that it has high GI (gastrointestinal) absorption and does not violate the Lipinski principle of drug-likeness.

For the hydrolysis reaction, the larger 6-311++G(d,p)/lanl2dz basis set is used to justify the choice of 6-31G(d)/lanl2dz basis set. Under the larger 6-311++G(d,p)/lanl2dz basis set, the obtained Gibbs free energy difference for hydrolysis reaction is 0.4 eV, and the activation energy barrier is also relatively small (0.493 eV), which shows approached energetic tendency as the results obtained at smaller 6-31G(d) basis set. Meanwhile, the smaller basis set at 6-31G(d)/lanl2dz can generate substantially accurate optimized structure for hydrolysis product (Cl-Pt and O-Pt bonds: 2.333 Å, 2.071 Å), which agrees very well with structure (Cl-Pt and O-Pt bonds: 2.336 Å, 2.067 Å) at larger basis set 6-311++G(d,p)/lanl2dz.

### 3.6. Interaction Profile of Pt(III) Compound with Purine Bases

The HOMO-LUMO gap of the guanine base is calculated to be 7.944 eV at M06-2X/6-311++G(p,d) theoretical level. The dipole moment is 6.41 Debye. Mulliken charge population analysis (Figure S4) demonstrates that the imino N7 nitrogen carries a smaller negative charge, −0.47 |e|, compared to other nitrogen atoms. Another double-bonded nitrogen (pyridinium N3) carries a negative charge of −0.58 |e|. Two secondary amine nitrogen atoms are negatively charged by −0.71 and −0.76 |e|. The amino nitrogen with chemical inertness carries the most damaging charge, −0.82 |e|. The nitrogen at the IS state is less charged than free purine (Table 3) because of the electron-withdrawing of spatially adjacent high electronegative nitrogen and chlorine atoms. The charge value of imino N7, which is the coordination site, is increased during the combination reaction from the IS state to the FS state. The electrostatic potential (ESP, Figure S4) contour shows that the electron accumulation is mainly reflected near the guanine’s imino nitrogen and carbonyl group. At the same time, the ESP images of other parts primarily exhibit electron depletion characteristics.

Frontier molecular orbital analysis shows that the initial state LUMO+1 empty orbital overlaps orbital components at the hydrogen bond position, while most of the transition state LUMO+1 components are distributed on guanine (Figure S5). For the coordination reaction products, LUMO+1 is almost entirely distributed on guanine. The initial and transition states of LUMO are mainly distributed in the platinum compound, indicating that empty orbits of the platinum compound have the potential to receive electron lone pairs from the guanine base. The transition state LUMO includes an obvious p-character orbital of N7. The product LUMO is mainly located at guanine. During the coordination reaction process, there are significant changes in the distribution of frontier molecular orbitals, with the HOMO of the initial state adduct primarily located on the purine base, providing the possibility for electron transfer to the empty orbitals of the platinum compound in the LUMO. For the transition state and product, the high-energy occupied orbitals HOMO and HOMO-1 mainly distribute on the platinum compound. In contrast to the initial state, the lower energy-occupied orbitals (HOMO-2 and HOMO-3, Figure S6) show significant distribution on the guanine ligand. The HOMO and HOMO-1 of the transition state and product are mainly distributed on the platinum compound, indicating clear electron transfer and orbital rearrangement.

**Figure 12:**
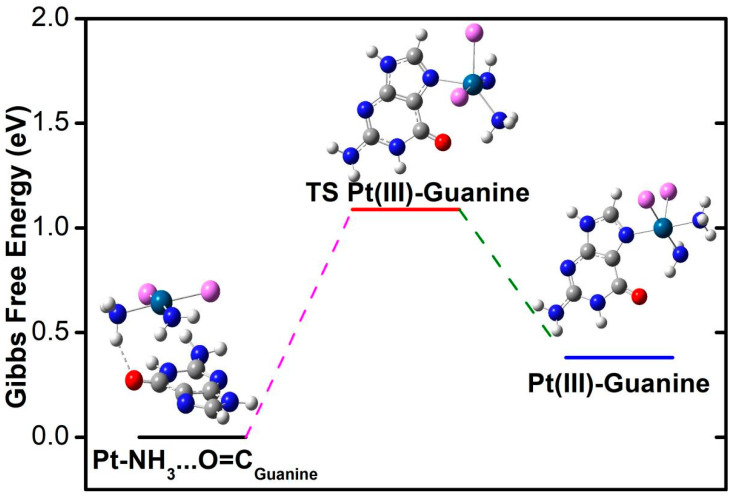
Potential energy diagram for guanine interaction with the Pt(III) compound. Relaxed structures for physical adduct with hydrogen bonding (initial state), transition state (TS), and the formation of coordination bond (final state).

As seen from the reaction Gibbs free energy plot (Figure 12), the coordination reaction of guanine with platinum compound requires an endothermic energy of 0.381 eV and an activation energy barrier of 1.088 eV. The hydrogen bond for the guanine and platinum compound shows a distance of 1.858 Å, and the NNClCl dihedral angle for the platinum compound plane is 171.6°. Upon reaching the transition state, the dihedral angle shrinks to 141.7°, reflecting the distortion of the planar structure of the platinum compound when they are close to the guanine base. The dihedral angle of NNClCl was further reduced to 106.8° at the final state. The infrared spectrum for fingerprint and characteristic regions are predicted in Figure S7 and the vibration mode variation as the reaction proceeds is discussed in conjunction with the figure.

For the combination reaction between platinum compound and guanine base, the Gibbs free energy difference and activation energy barrier at 6-311++G(d,p)/lanl2dz basis set are calculated to be 0.397 eV and 1.099 eV, which is very close to the energetic parameters obtained at 6-31G(d)/lanl2dz basis set (0.381 eV and 1.088 eV). In addition, the structural parameters obtained at 6-31G(d)/lanl2dz and 6-311++G(d,p)/lanl2dz agree very well (as shown in Figure S8). For example, at 6-311++G(d,p)/lanl2dz basis set, the N(guanine)-Pt, Cl-Pt coordination bonds and total dipole moments of the combination reaction product are 2.016 Å, 2.445 Å and 10.14 Debye, which are consistent with the parameters obtained at 6-31G(d)/lanl2dz basis set (2.058 Å, 2.451 Å and 10.33 Debye). Thus, the 6-31G(d)/lanl2dz should be reliable for predicting platinum compound and guanine’s structural and reaction properties.

**Figure 13:**
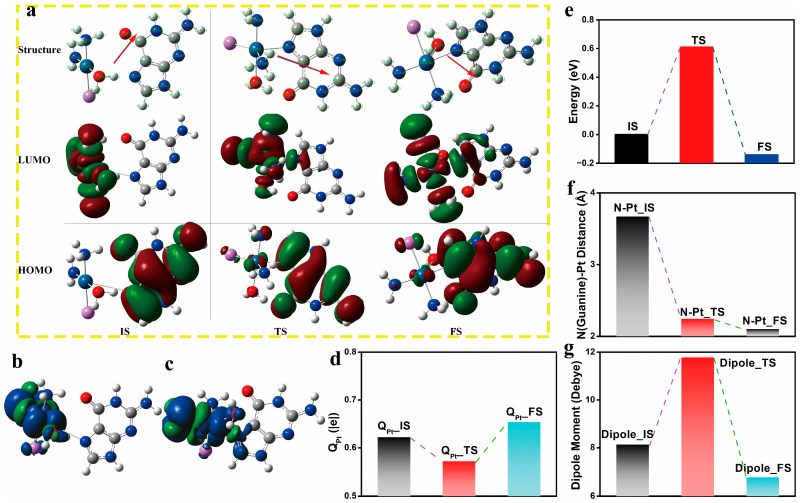
The optimized structures with dipole moment vectors (shown in red arrows), LUMO and HOMO frontier orbitals for reactant (IS), transition state (TS), and final product (FS, (**a**)); the electron spin density for reactant (**b**) and final product (**c**); the platinum NBO charge population (**d**), enthalpy diagram (**e**), N(Guanine)-Pt distance (**f**) and dipole moment analysis (**g**). The isosurface values for molecular orbital and electronic density are 0.02 and 0.0004, respectively.

The coordination reaction of guanine with the hydrolytic platinum compound to form the product (Figure 13) is exothermic by −0.158 eV, and the activation energy barrier required for the reaction is 0.647 eV in the scheme of Gibbs free energy. As can be seen from the arrows in Figure 13a, the direction of the dipole moment vector of the reaction system is from the platinum compound to guanine. The dipole moment is defined as the expression,

(6) $$\vec{D}=\sum_{i}q_{i}\left(r_{i}^{-}-r_{i}^{+}\right)=\underset{i}{\sum }q_{i}\vec{d_{i}}$$

where $\vec{d_{i}}$ is the position vector from the positive to the negative charge center. Thus, the calculated dipole moment vector suggests that negative and positive charge centers are located at guanine and platinum compounds, respectively. In terms of frontier molecular orbitals, the reactant consists of an admixture containing two hydrogen bonds: the hydrogen bond between the amino hydrogen and the carbonyl oxygen (1.828 Å) and the hydrogen bond between water hydrogen and the imidazole nitrogen (1.546 Å). The HOMO of the initial adduct is all distributed on guanine, and the HOMOs of the transition state structure and the product structure are composed of contributions from both guanine and platinum compound. The LUMO of the initial adduct is all distributed in the platinum compound. At the same time, the LUMOs of the transition state and the product structure are jointly contributed by guanine and the platinum compound, indicating that the contribution of guanine to LUMO increases as the reaction proceeds. After the reaction, the LUMO energy level of the whole system increased from −3.663 eV to −3.619 eV, and the HOMO decreased from −10.476 eV to −10.645 eV. The HOMO-LUMO energy gap is increased, hinting at the enhanced stability of the coordination product. The results of the orbital analysis showed that the frontier molecular orbitals of guanine and platinum compounds overlapped effectively during the reaction. The spin density of the initial state of the hydrogen-bonded admixture is mainly distributed on the platinum compound.

In contrast, the spin density of the final state structure is extended to the imidazole ring of the purine base (Figure 13). As the reaction progresses, the imidazole nitrogen of guanine gradually approaches the central ion of Pt, and the N-Pt distance shrinks from 3.672 Å to 2.103 Å. The charge of the central platinum ion decreases from 0.62 |e| in the initial state to 0.57 |e| in the transition state and then increases to 0.65 |e| in the final state. The dipole moment of the system increases from 8.14 Debye in the initial state to 11.78 Debye in the transition state. Then, it drops sharply to 6.77 Debye in the product, indicating that the charge separation degree of the system increases at the initial stage of the reaction, and the charge separation and polarization decrease after the formation of the product.

### 3.7. Influence of the Temperature and the Substituting Group

**Figure 14:**
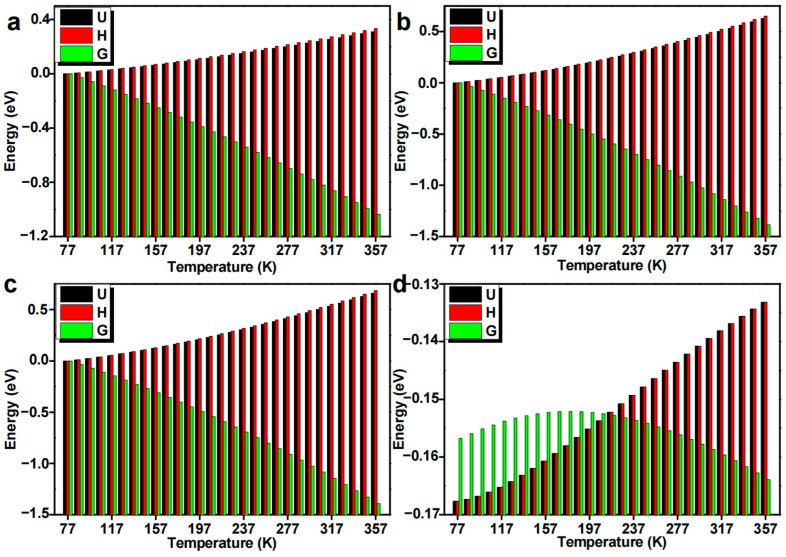
Temperature influence on the energetics (*U, H, G*) of primary platinum hydrolysis species (**a**), the initial reactant adduct (**b**), the coordination product (**c**), and the coordination reaction energy of primary platinum hydrolysis species interacting with guanine (**d**).

We have discussed the temperature effects for platinum hydrolysis species, the initial reactant adduct, the coordination product, and the coordination reaction energy of primary platinum hydrolysis species interacting with guanine (Figure 14). The temperature effect for the primary hydrolysis species at 1 atm constant pressure is shown in Figure 14. The considered temperature range is from liquid nitrogen temperature 77 K to critical temperature for protein denaturation 357 K. It can be found from the calculation results that the thermal dynamic energy (*U*) and enthalpy (*H*) increase with the rising temperature. Nevertheless, the Gibbs free energy (*G*) decreases and becomes more negative with increasing temperature. The adduct between hydrolysis species and guanine base shows a more significant energetic variation tendency with rising temperature.

**Figure 15:**
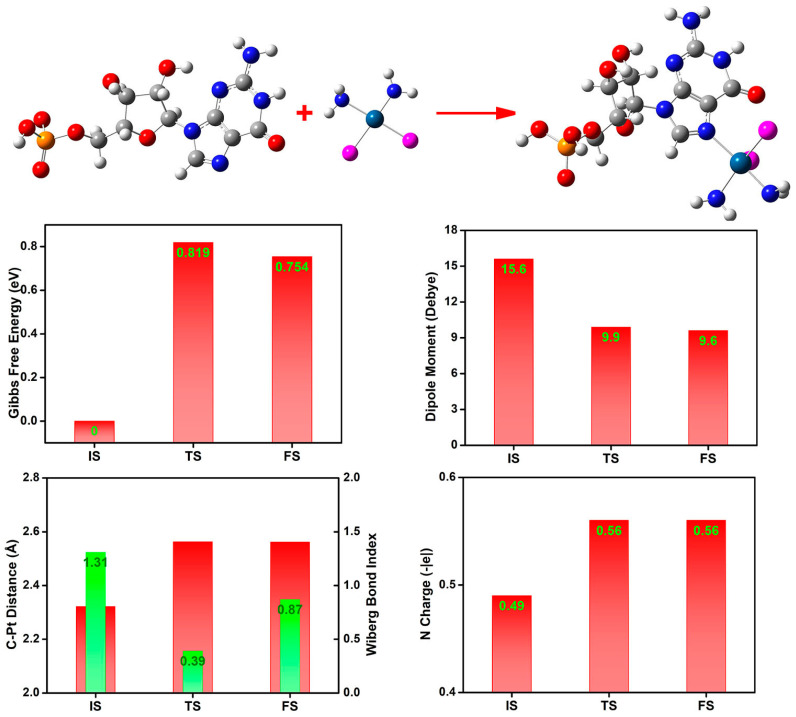
The energetics, dipole moment, Cl-Pt distance, Wiberg bond index, and N(5′-guanylic acid) Mulliken charge during coordination reaction between 5′-guanylic acid and platinum compound.

When the substituting phosphorous group is included in the coordination reaction, we employ 5′-guanylic acid as the reactant (Figure 15), a typical purine nucleotide and a vital DNA and RNA building block. The Gibbs free energy difference is 0.754 eV, and the activation barrier is 0.819 eV. Thus, the substituting phosphorous groups favor the coordination reaction with the platinum compound, lowering the guanine-Pt combination’s activation barrier. The 5′-guanylic acid presents an N charge of penta-heterocycle of 0.49 |e| at the isolated state, while at the TS and FS states, the corresponding coordinating nitrogen is more negatively charged (−0.56 |e|). During the coordination reaction, the Pt-Cl distance is significantly lengthened from 2.322 to 2.562 Å, and the charge of platinum is decreased from 0.39 to 0.34 |e|. The Wiberg bond index of Cl-Pt at the reaction site is 1.31, 0.39, and 0.87 for IS, TS, and FS states. Thus, one Cl-Pt bond should be partially broken during the coordination process. The dipole moment is decreased during the reaction, indicating that the charge separation should be suppressed sectionally due to the charge transfer.

## 4. Conclusions

Through density-functional theory calculations, this contribution reveals the formation, stability, chemical adsorption, and activation effects of cisplatin on the gold–magnesia composite structure. The mechanisms of the hydrolysis reaction of the activated platinum compound and its binding mechanism with DNA base are studied. Compared to the interface structure formed by Mg approaching Au, the heterojunction structure with vertical O-Au bonding interaction is more favorable, with an energy reduction of 1.709 eV. Differential charge density indicates extensive charge transfer at the oxide–metal interface, with a yellow band of charge accumulation observed at the interface region, suggesting adequate bonding between magnesium oxide and gold. Cisplatin exhibits electron acceptor characteristics when forming Pt(III) and Pt(IV) states on the magnesium oxide film, with negative charges of −0.34 and −0.58 |e|. The higher the oxidation state of Pt(II to IV), the more electrons transfer from the Au-MgO composite material to the platinum compound. The Pt-N distance of the adsorbed cisplatin is shorter than that of free cisplatin, indicating strengthened 5d←2p coordination bonds. For the flat and oblique adsorption modes of cisplatin, there is no significant difference in the density of states of the gold and magnesium oxide film structures, indicating the maintenance of the heterojunction structural framework. However, there are substantial changes in the electronic states of cisplatin itself with different adsorption configurations. In the flat configuration, the band gap width from the top of the valence band to the bottom of the conduction band of cisplatin is larger than that of the oblique configuration. The Cl-Pt bond range in the Pt(III) compound shows apparent charge reduction on the magnesium oxide film, indicating that this Cl-Pt bond is an active site with potential for decomposition and hydrolysis.

Substitution of chloride ions by water molecules can lead to hydrolysis products of the platinum compound, enhancing the polarization of the magnesium oxide–gold composite material and showing strong charge separation. Due to the strong adsorption of chloride ions, primary hydrolysis of platinum compound is a slightly exothermic process with an energy decrease (−0.189 eV). Hydrolysis of the free compound is endothermic by 0.309 eV, requiring crossing an activation energy barrier of 0.399 eV, indicating that hydrolysis of this platinum(III) compound is easily achievable. Secondary hydrolysis is an unfavorable endothermic process (0.539 eV), requiring crossing an activation energy barrier of 1.006 eV. ADME prediction parameters indicate that the hydrolysis product is consistent with Lipinski’s rule and has good ESOL solubility and high GI absorption. During the coordination reaction process, there are significant changes in the distribution of frontier molecular orbitals, with the HOMO of the initial state adduct primarily located on the purine base, providing the possibility for electron transfer to the empty orbitals of the platinum compound in the LUMO. The HOMO and HOMO-1 of the transition state and product are mainly distributed on the platinum compound, indicating clear electron transfer and orbital rearrangement. Direct coordination reaction between purine and Pt(III) compound requires an endothermic reaction of 0.381 eV, with an activation energy barrier of 1.088 eV. The activation energy barrier for purine coordination reaction with hydrolysis products is reduced to 0.647 eV, and the dipole moment gradually decreases to 6.77 Debye during the reaction, indicating a reduction in the system’s charge separation and polarization. The substituting phosphorous groups favor the coordination reaction with the platinum compound. The adduct between hydrolysis species and guanine base shows a more significant energetic variation tendency with rising temperature.

The cisplatin-based platinum compounds are essential clinical chemotherapeutic agents that participate in most tumor chemotherapy regimens. The activation of cisplatin is important for its easy hydrolysis, which is the crucial primary step before binding DNA. The theoretical calculation results are helpful for understanding the hydrolysis of and its combination with DNA purine base. Low blood magnesium can cause a range of conditions, such as nausea, vomiting, anorexia, muscle weakness, seizures, and even coma. Platinum-based drugs are used in combination with magnesium oxide. They are thought to reduce the neurotoxicity and nephrotoxicity of platinum-based drugs, as well as the prevalence of hypomagnesemia caused by platinum-based drugs, with potential clinical value. This study is expected to provide theoretical clues and data references for developing inorganic oxide carriers for cisplatin drugs.

## Figures and Tables

**Table 1:** Bader charges population analysis for cisplatin adsorption on gold (111)-supported magnesia (111) film (unit in electron).

	Isolated State	Flat Adsorption	Pt(III) State	Pt(IV) State
Cisplatin	0	−0.11	−0.34	−0.58
NH_3_/NH_2_ ^a^	+0.22, +0.23	+0.26, +0.26	+0.25, −0.45	−0.42, −0.45
Cl	−0.49, −0.50	−0.55, −0.57	−0.58, −0.59	−0.62, −0.64
Pt	+0.54	+0.50	+0.45	+0.43
MgO	0	+2.58	+2.83	+3.02
Au	0	−2.47	−2.50	−2.45

^a^ For Pt(III) compound, the ligands are NH_3_ and NH_2_. For the Pt(IV) compound, the ligands are two NH_2_.

**Table 2:** Structural parameters for Pt(III) compound compared with cisplatin Pt(II) compound on Au(111)-MgO(111) composite. Units for bond distance and angles are in Å and degrees, respectively.

	Pt(III) Compound	Free Cisplatin	Adsorbed Cisplatin
Pt-N distance	2.073, 2.055	2.092	2.061, 2.066
Pt-Cl distance	2.367, 2.343	2.288	2.329, 2.334
N-Pt-N angle	91.7	98.7	94.7
Cl-Pt-Cl angle	92.1	95.3	92.0
Mg-Cl distance	2.492, 2.658		2.478, 2.549

**Table 3:** Mulliken charge population analysis for nitrogen for free purine and the purine nitrogen during a combination reaction (IS, TS, FS as shown in Figure 12) with platinum ion (unit in electron).

	Free Purine	IS	TS	FS
imino N7	−0.47	−0.41	−0.54	−0.61
pyridinium N3	−0.58	−0.46	−0.48	−0.48
secondary amine N1, N9	−0.76, −0.71	−0.68, −0.67	−0.74, −0.68	−0.73, −0.68
exocyclic amino nitrogen	−0.82	−0.79	−0.81	−0.81

## Data Availability

The original data of this contribution are included in the article/Supplementary Material. Further inquiries can be directed to the corresponding author.

## Supplementary Materials

The following supporting information can be downloaded at: https://www.mdpi.com/article/10.3390/nano14242027/s1, Figure S1: Interlayer gold-gold bond distance and the printed d electrons after relaxation of gold substrate; Figure S2: The optimized structure and surface rumpling value for gold-supported two monolayer magnesia (111), which show much larger surface distortion than one monolayer magnesia; Figure S3: The influence of solvent effect on the dipole moment (blue line), N-Pt and Cl-Pt bonding distance, and the NBO charge population; Figure S4: Mulliken charge population and dipole moment vector, and electrostatic potential (ESP) contour for guanine molecule; Figure S5: The frontier molecular orbitals for guanine reacting with Pt(III) compound; Figure S6: Lower energy-occupied orbitals HOMO-2 and HOMO-3; Figure S7: Fingerprint regions (left panel) and characteristic regions (right panel) of infrared spectrum; Figure S8: The structural parameters obtained at 6-31G(d)/lanl2dz and 6-311++G(d,p)/lan2dz basis. Table S1: The optimized structural and electronic parameters at OptB88-vdW and PBE functionals; Table S2: Thickness examination of interlayer gold-gold bond distance and the printed d electrons [71,72,73].
