# Supplementary material for: Platinum Compound on Gold–Magnesia Hybrid Structure: A Theoretical Investigation on Adsorption, Hydrolysis, and Interaction with DNA Purine Bases
Source: Nanomaterials (Basel). 2024 Dec 17;14(24):2027. doi: 10.3390/nano14242027 (PMC11678598; doi:10.3390/nano14242027)
Supplement: Supplementary file 1 [file nanomaterials-14-02027-s001.zip › nanomaterials-3335756-supplementary.pdf]

Supporting Information

Platinum Compound on Gold–Magnesia Hybrid Structure: A Theoretical Investigation on Adsorption, Hydrolysis, and Interaction with DNA Purine Bases

Zhenjun Song [1,*], Mingyue Liu [1], Aiguo Zhong [1], Meiding Yang [1], Zhicai He [1], Wenmin Wang [3] and Hongdao Li [2,*]

[1] Engineering Research Center of Recycling & Comprehensive Utilization of Pharmaceutical and Chemical Waste of Zhejiang Province, School of Pharmaceutical and Chemical Engineering, Taizhou University, Taizhou 318000, China; liumingyue0820@126.com (M.L.); zhongaiguo@tzc.edu.cn (A.Z.); yangmd@tzc.edu.cn (M.Y.); hezhicai@tzc.edu.cn (Z.H.)

[2] Department of Chemistry and Chemical Engineering, Taiyuan Institute of Technology, Taiyuan 030008, China

[3] College of Chemistry and Materials, Taiyuan Normal University, Jinzhong 030619, China; wangwenmin0506@126.com

* Correspondence: songzj@mail.nankai.edu.cn (Z.S.); lihong.dao@163.com (H.L.)

S1. The optimized structural and electronic parameters at OptB88-vdW and PBE functionals

Table S1 The optimized O-Mg bond length (in Å), lattice constant (in Å) and band gap (in eV) of bulk magnesia at OptB88-vdW and PBE functionals

	OptB88-vdW	PBE	Expt.[a]
O-Mg distance	2.116	2.122	2.11
lattice constant	4.232	4.244	4.22
band gap	5.118	4.506	5.4

a. The experimental O-Mg distance and lattice constant are from reference [1]. The experiment band gap is optical band gap measured using UV diffused reflectance spectroscopy[2].

Table S2 The structural parameters (distance in Å and angle in degree) of cisplatin obtained at OptB88-vdW, PBE functional and hybrid functional M06-2X

	OptB88-vdW	PBE	M06-2X
Cl-Pt distance	2.289	2.284	2.339
N-Pt distance	2.093	2.085	2.116
Cl-Pt-Cl angle	95.3	95.7	95.4

S2 Thickness examination of interlayer gold-gold bond distance and the printed d electrons

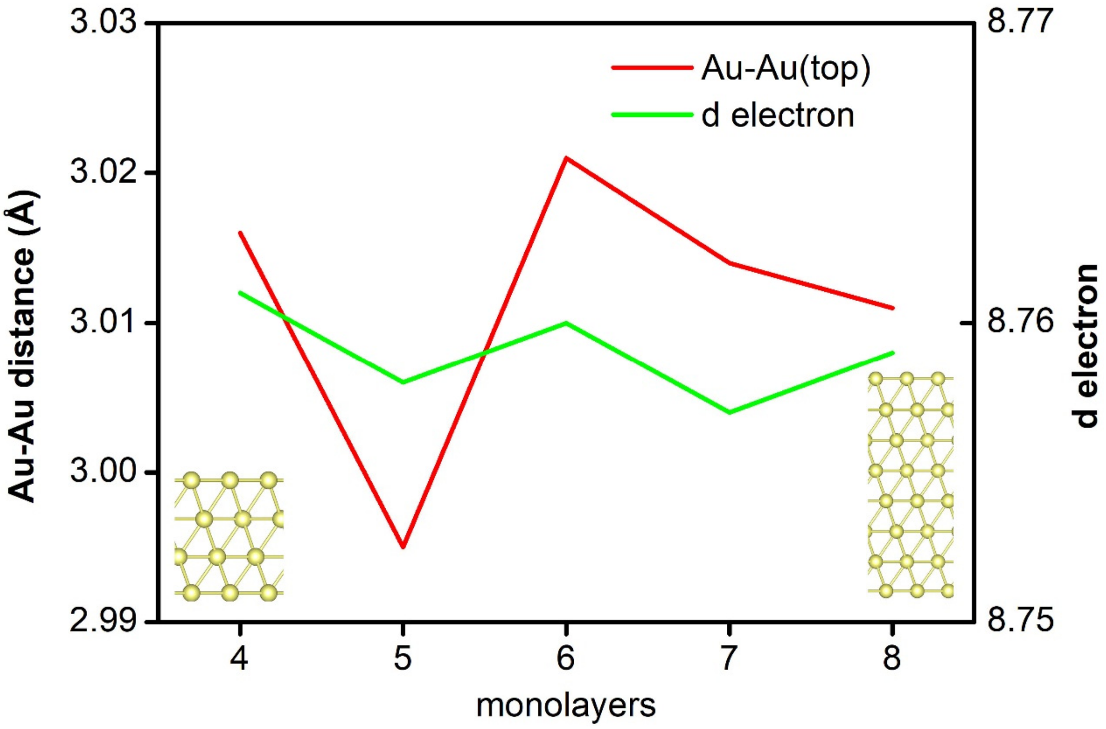

Figure S1 Interlayer gold-gold bond distance and the printed d electrons after relaxation of gold substrate.

S3. Optimized structure and surface rumpling value for gold-supported two monolayer magnesia

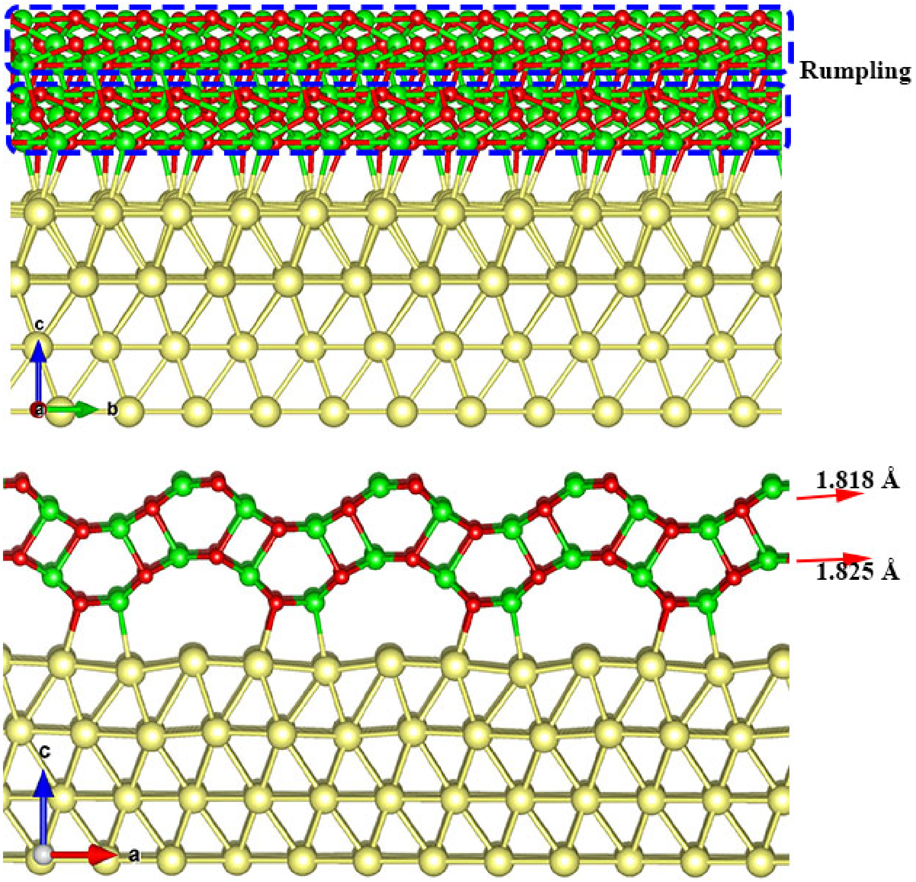

Figure S2 The optimized structure and surface rumpling value for gold-supported two monolayer magnesia (111), which show much larger surface distortion than one monolayer magnesia. The large distortion of magnesia (111) agrees with the experimental metal-supported magnesia (111) morphology showing extended slight corrugation [3-5].

S4 The influence of solvent effect on the dipole moment, bonding distance and the charge population

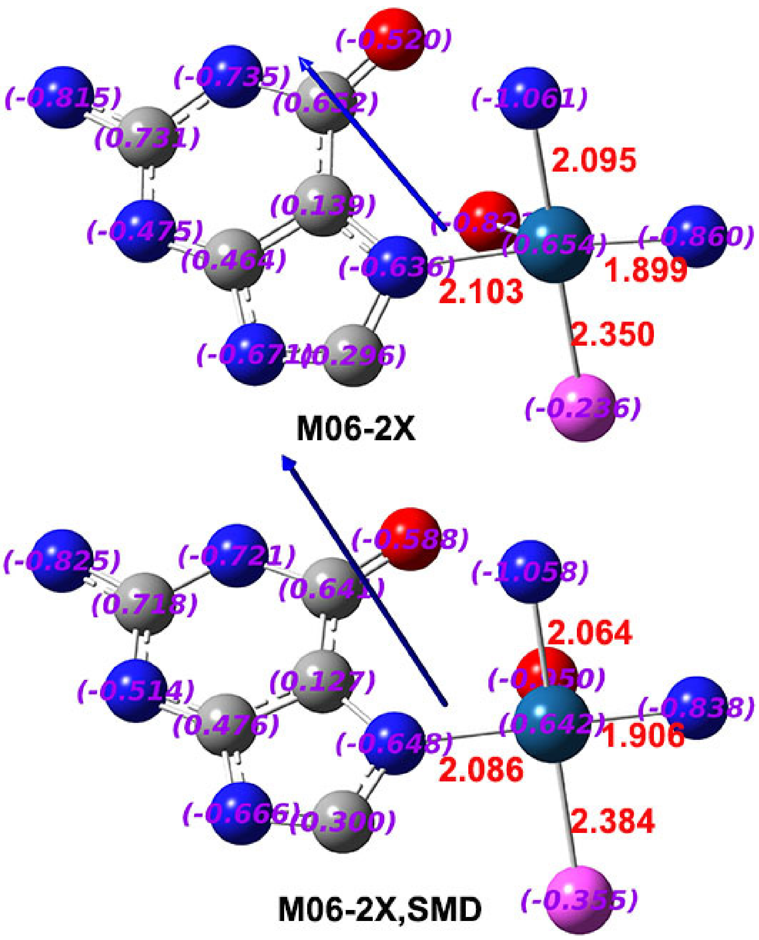

Figure S3 The influence of solvent effect on the dipole moment (blue line), N-Pt and Cl-Pt bonding distance (red color), and the NBO charge population (purple color in paratheses). The adduct in bottom panel are optimized using SMD (Solvation Model based on Density) hidden solvent model [6, 7].

S5 Mulliken charge population and dipole moment vector, and electrostatic potential (ESP) contour for guanine molecule.

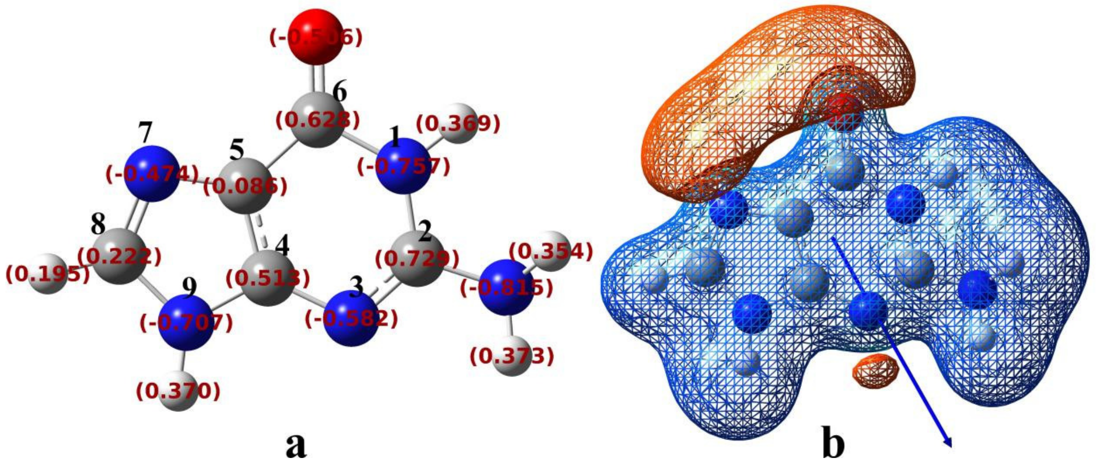

Figure S4 Mulliken charge population (a), dipole moment vector and electrostatic potential (ESP) contour (b) for guanine molecule. The isosurface value for mapped ESP is 0.05. The red and blue colors denotes negative and positive potential respectively.

S6 The frontier molecular orbitals for guanine reacting with Pt(III) compound

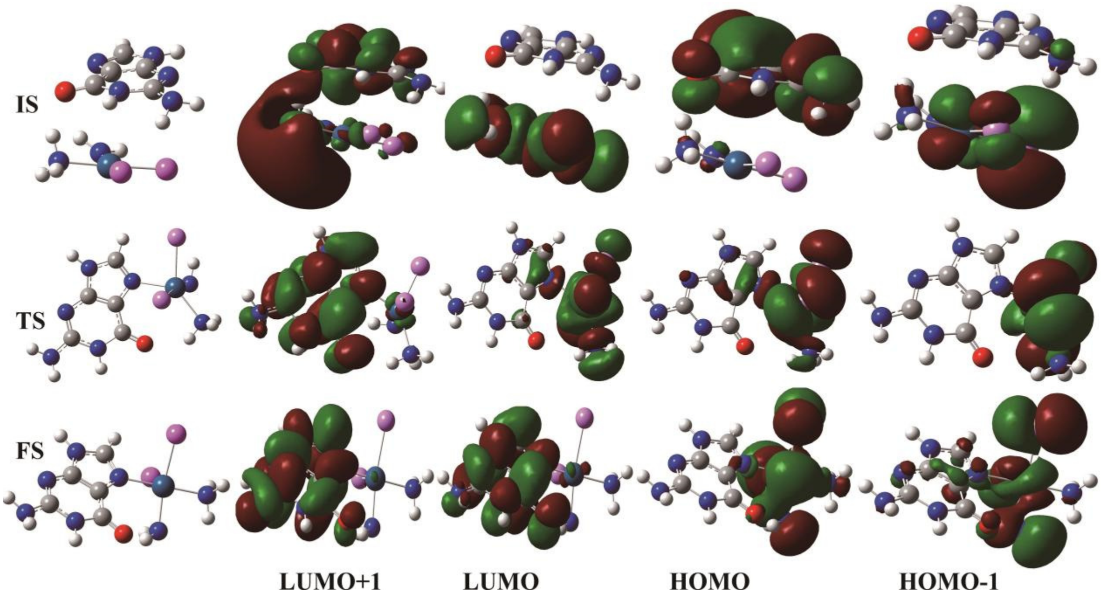

Figure S5 The frontier molecular orbitals for guanine reacting with Pt(III) compound with isosurface value of 0.02: initial state (top panel), transition state (middle panel), final state (bottom panel). The gray, blue, white, purple and cyan colored balls represent carbon, nitrogen, hydrogen, chlorine and platinum, respectively.

S7 Lower energy occupied orbitals HOMO-2 and HOMO-3

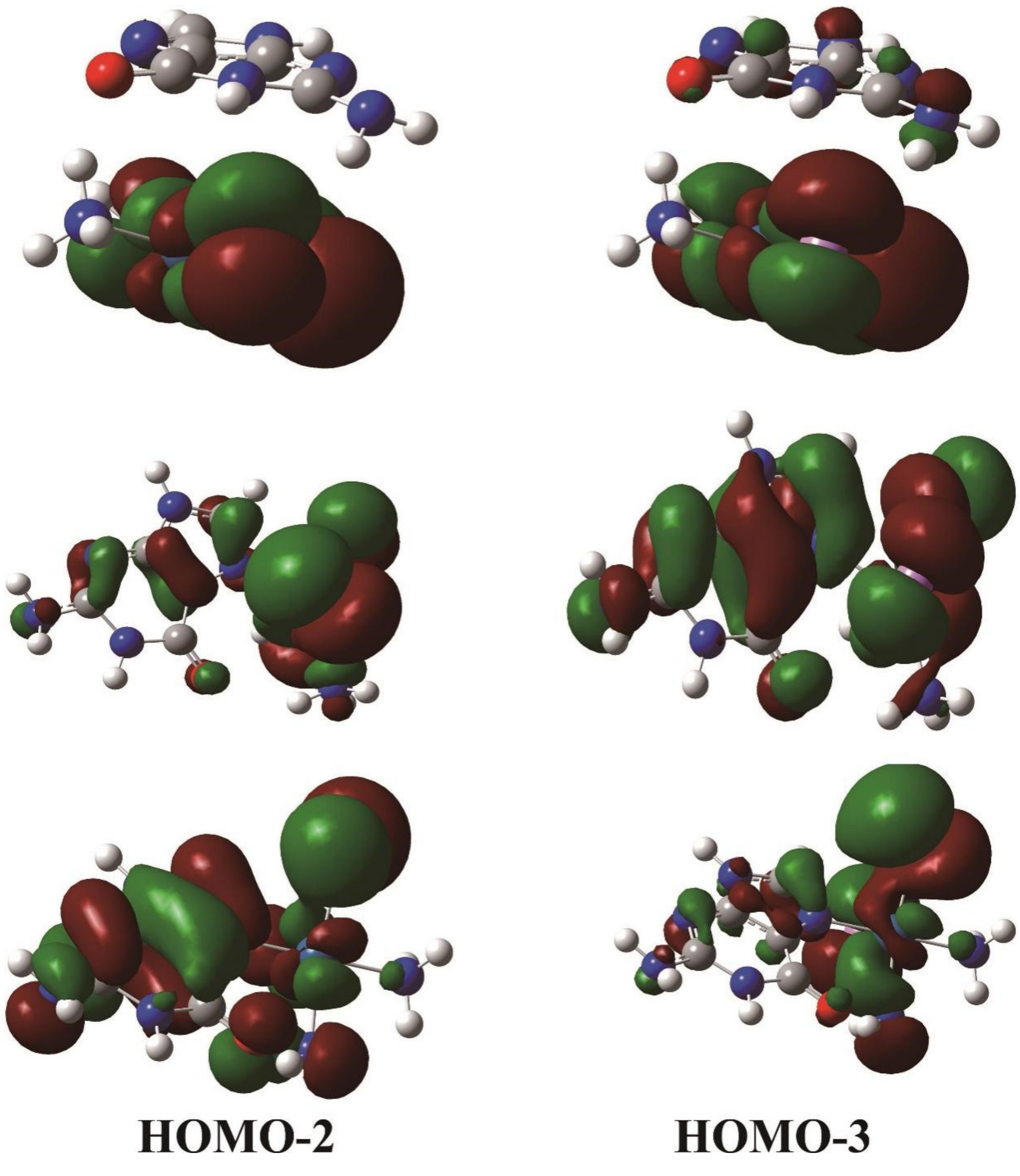

Figure S6 Lower energy occupied orbitals (HOMO-2 and HOMO-3) for IS (top), TS (middle) and FS (bottom) for coordination reaction between guanine and activated platinum compound.

S8 Discussion for predicted infrared spectrum during coordination reaction between platinum compound and the guanine base

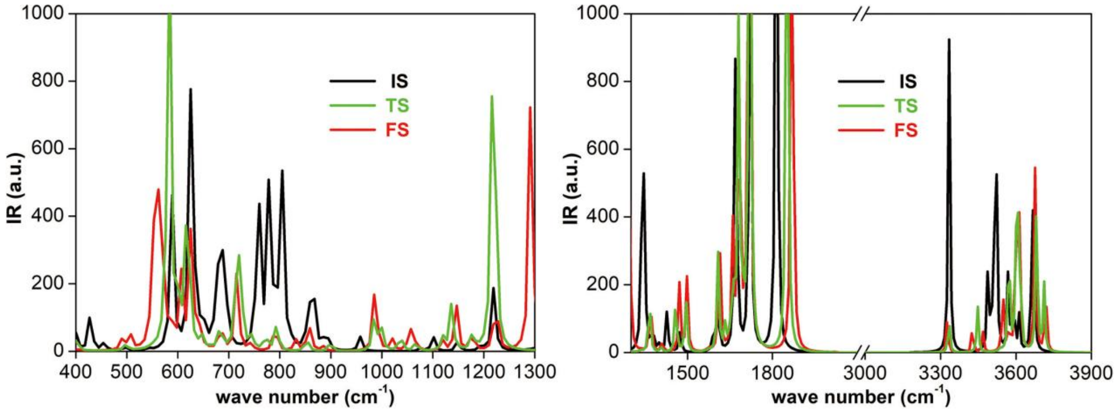

Figure S7 Fingerprint regions (left panel) and characteristic regions (right panel) of infrared spectrum.

Discussion for predicted infrared spectrum during coordination reaction between platinum compound and the guanine base:

The infrared fingerprint region indicates the absorption peaks of 758, 780, 803 cm^{-1} at initial state, which can be attributed to the out-of-plane bending vibration of N-H of the six-membered N-heterocycle, the out-of-plane rocking vibration (ω) of the substituted amino N-H, and the out-of-plane bending vibration (γ) of the guanine backbone, respectively, are greatly attenuated (Fig. 15). For the fingerprint region of the five-membered heterocyclic ring, N9-H out-plane bending vibration of the initial adduct structure is 589cm^{-1}, while the frequency of this vibration mode of the transition state is red shifted to 583 cm^{-1},

and the corresponding N9-H of the product doesn't exhibit out-of-plane bending vibration at nearby frequencies. The five membered heterocycle is coordinated to the Pt central ion, and the degree of freedom is greatly reduced, thus N9-H bending vibration of the product is quenched. The amino group of product shows out-plane bending vibration (γ) at 565 cm^{-1}. In addition, compared to the initial adduct reactant, the product shows out-plane twisting vibration (τ) at 429 cm^{-1}. The product shows strong absorption peak at 1292 cm^{-1} and 1870 cm^{-1}, assigned to symmetric deformation vibration (δ^s) of ammonia molecule and symmetric stretching vibration (V^s) of carbonyl group. After the coordination of the platinum compound with guanine, the C-H symmetric stretching vibration peak (3327 cm^{-1}) of the five-membered heterocyclic ring near the coordination site is blue shifted and its intensity is increased from 0.1 to 33 compared with the vibration peak (3307 cm^{-1}) of the initial state of the adduct reactant. After guanine coordination reaction, the N-H symmetrical stretching vibration peak with high intensity of the azocyclic ring is blue shifted from 3670 cm^{-1} in the initial structure to 3676 cm^{-1} in product. The N-H asymmetric stretching vibration of the secondary amine NH_2 group blue shifts from 3663 cm^{-1} in the initial structure to 3712 cm^{-1} in the transition state, and then blue shifts again to 3718 cm^{-1} in the product as the reaction proceeds.

S9 The structural parameters obtained at 6-31G(d)/lanl2dz and 6-311++G(d,p)/lan2dz basis sets

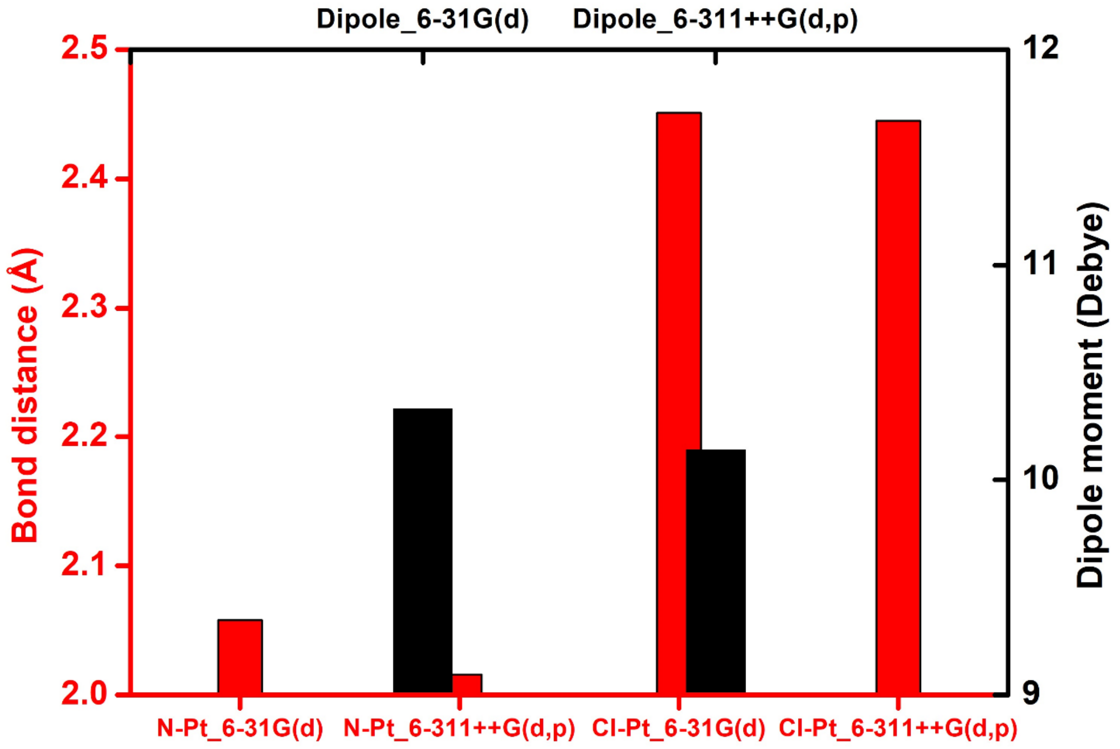

Figure S8. The structural parameters N(guanine)-Pt, Cl-Pt and dipole moment obtained at 6-31G(d)/lanl2dz and 6-311++G(d,p)/lan2dz basis sets.

References

[1] P. Karen, A. Kjekshus, Q. Huang, V.L. Karen, The crystal structure of magnesium dicarbide, J. Alloys Compd., 282 (1999) 72-75.

[2] K. Mageshwari, S.S. Mali, R. Sathyamoorthy, P.S. Patil, Template-free synthesis of MgO nanoparticles for effective photocatalytic applications, Powder Technol., 249 (2013) 456-462.

[3] B. Sarpi, R. Daineche, C. Girardeaux, A. Hemeryck, S. Vizzini, Ultra-thin MgO(111)-polar sheets grown onto Ag(111), Appl. Surf. Sci., 361 (2016) 259-264.

[4] M. Yeganeh, F. Kafi, Stability and thermoelectric properties of the MgO monolayers under tensile and compressive strain, Physica E, 123 (2020) 114176.

[5] M. De Santis, V. Langlais, K. Schneider, X. Torrelles, Growth-mode and interface structure of epitaxial ultrathin MgO/Ag(001) films, J. Phys.: Condens. Mat., 33 (2021) 265002.

[6] S. Miertuš, E. Scrocco, J. Tomasi, Electrostatic interaction of a solute with a continuum. A direct utilizaion of AB initio molecular potentials for the prevision of solvent effects, Chem. Phys., 55 (1981) 117-129.

[7] A.V. Marenich, C.J. Cramer, D.G. Truhlar, Universal Solvation Model Based on Solute Electron Density and on a Continuum Model of the Solvent Defined by the Bulk Dielectric Constant and Atomic Surface Tensions, J. Phys. Chem. B, 113 (2009) 6378-6396.
